# Construction of A Triple-Stimuli-Responsive System Based on Cerium Oxide Coated Mesoporous Silica Nanoparticles

**DOI:** 10.1038/srep38931

**Published:** 2016-12-12

**Authors:** Jia Wen, Kui Yang, Yongqian Xu, Hongjuan Li, Fengyu Liu, Shiguo Sun

**Affiliations:** 1 Shaanxi Key Laboratory of Natural Products & Chemical Biology, College of Science, Northwest A&F University, Yangling, Shaanxi 712100, People’s Republic of China; 2 State Key Laboratory of Fine Chemicals, School of Chemistry, Dalian University of Technology, No.2 linggong Road, Ganjingzi, District, Dalian 116023, People’s Republic of China

## Abstract

In this work, a triple-stimuli (GSH, pH and light irradiation) responsive system were designed based on CeO_2_ nanoparticles (CeO_2_ NPs) coated doxorubicin (DOX) and photosensitizer hematoporphyrin (HP) dual-loaded mesoporous silica nanoparticles (MSN). Upon entering into cancer cells, both high concentration of intracellular GSH and low pH environment would reduce CeO_2_ NPs to cerium ions, accompanied with the degradation of CeO_2_ NPs and the conformational change of HP under light irradiation, the preloaded DOX are thus released from the nanocarrier, resulting in a contrast fluorescence enhancement. Meanwhile, ^1^O_2_ generated from HP for potential photodynamic therapy (PDT) upon light irradiation. In comparison, not much influence can be observed for normal cells. This nanosystem not only has a significantly enhanced efficacy for cancer cells but also broad the scope for the future design and applications of multifunctional platforms for synergetic chemotherapy and PDT.

Chemotherapy remains as a major treatment modality for cancer nowadays^1^. To improve its efficiency and minimize the adverse effects, controlled stimuli-responsive nanosystem for on-demand drug release is highly desirable^2^. In recent years, a lot of researches have been carried out on stimuli-responsive systems^3^,^4^,^5^,^6^,^7^. An ideal stimuli-responsive nanosystem should have the following characteristics: (i) recognize tumor microenvironment in high selective manner; (ii) allow for precise release in response to exogenous or endogenous stimulus. Initial, stimuli-responsive systems was conducted by various single control, including pH^8^,^9^,^10^,^11^,^12^, redox potential^13^,^14^,^15^,^16^,^17^, temperature^18^, biomolecules^19^, light^20^, etc. For example, Tian and co-workers designed and synthesized mesoporous silica nanoparticles (MSN) with pH sensitive valves that can deliver and release the anti-cancer doxorubicin (DOX) to tumor cells in a pH dependent switch on/off status^21^. However, most of them are easily affected by complex external factors and suffered the problem of low release accuracy and some side effects. To solve this problem, dual-stimuli release systems have been developed via the combination of two kinds of stimulus^22^,^23^,^24^. For example, Chen and Liu conducted hyaluronic acid (HA) and PAMAM dendrimer (PAMAM-G2) to cap dual drug-loaded MSN and the precise release of drugs could be triggered by two intracellular stimuli, a low pH value and glutathione^24^. Lately, for more precise release in complicated blood circulation and pathological environment, triple even multiple stimuli-responsive nanosystems have been developed^25^,^26^,^27^,^28^,^29^,^30^,^31^,^32^.

Although lots of efforts have been made, yet the efficacy of chemotherapy is limited by the evolution of drug-resistant tumors after prolonged treatments^1^. Under this circumstance, synergetic therapy creates the possibility to solve the problem. Among them, photodynamic therapy (PDT) which employs exogenously produced reactive oxygen species (ROS) to kill cancer cells has been extensively exploited as a promising strategy for cancer cell killing and tumour ablation over the past decades^33^,^34^, and porphyrin derivatives are typical photosensitizer candidates used in PDT^35^. Unfortunately, most of them suffer from aggregation in aqueous solution due to π–π stacking and the hydrophobic effect of large π-conjugated molecular structures^36^,^37^, especially, not any specific interaction exists between the porphyrin molecules and the cancerous cells or tissues, leading to destructive side effects on the corresponding normal cells or tissues during PDT. Thus, to reach efficient overall therapy outcome, it is highly desirable to develop a stimuli-responsive nanosystem for precise release of anticancer drug like DOX and solve the above mentioned HP related PDT problem simultaneously.

To date, noninvasive and biocompatible MSN have been widely employed as a promising and attractive candidate for stimuli-responsive drug delivery system (DDS) owing to their tunable pore size, unique porous structure, high specific surface area, good biocompatibility and easy surface functionalization^38^,^39^,^40^,^41^. The ordered pore network of these MSN can entrap DOX drug and HP photosensitizer within the pores. Importantly, the pores could be gated with various gatekeepers, such as nanoparticles^42^, organic molecules^43^, and biomacromolecules^44^,^45^, to trigger the release of the entrapped drug in the presence of external or internal stimuli^46^,^47^. In 2015, Zhao’s group developed a pH, reduction and light triple-responsive nanocarriers based on poly(2-(diethylamino)-ethyl methacrylate) (PDEAEMA) modified hollow mesoporous silica nanopaicles (HMSNs). The linkages between HMSNs and pH-sensitive PDEAEMA polymer caps were composed of reduction cleavable disulfide bond and light-cleavable o-nitrobenzyl ester. Once the obtained drug-loaded HMSNs entering into tumor cells, the linkages would break up and the loaded drug could be released into the cytoplasm in acid or reduction intracellular environment. Moreover, the drug release could be further enhanced by external UV irradiation^48^.

It is known that the intracellular environment such as the concentration of GSH and the pH etc. is quite different in cancer and normal cells, and these factors can be employed for precise stimuli-response for further minimizing the external interferences and enhancing release efficiency and accuracy, especially for not affording any influence on the normal cells^8^,^9^,^15^,^16^. While, in reduction environment, cerium oxide nanoparticles (CeO_2_ NPs) can be converted to cerium ions^49^,^50^. Moreover, previous studies have demonstrated its biocompatibility and low or none toxicity^51^,^52^. Interestingly, CeO_2_ NPs also has strong fluorescence quenching ability^53^, which can be employed to efficiently quench the fluorescence of the loaded components and then restored during releasing, providing further fluorescence off-on evidence on the corresponding stimuli-responsive process. Taken together, CeO_2_ NPs can be a good gatekeeper candidate with exceptional antioxidant properties^54^,^55^,^56^,^57^, these features make it possible to construct a CeO_2_ NPs-MSN based multifunctional stimuli-responsive nanosystem.

Herein, as shown in Fig. 1, we have designed a novel nanosystem based on CeO_2_ coated, DOX-HP co-loaded MSN (both of HP and DOX have conjugation structures and they can combine through π-π interactions, this will be quite helpful for enhancing the loading efficiency of DOX into the MSN^58^) for intracellular triple-stimuli (reduced glutathione (GSH), pH and light irradiation) responsive release, potential synergetic therapy and enhanced therapeutic effect. When the as-prepared nanosystem entering into cancer cells, both high concentration of intracellular reduced GSH and low pH environment would reduce CeO_2_ NPs to cerium ions. And meanwhile upon light irradiation, the conformational change of HP would destroy the combination of DOX-HP^58^, not only leading to some synergetic curing effect due to the controlled-release of DOX and generation of ^1^O_2_ simultaneously, but also providing further fluorescence off-on evidence on the corresponding stimuli-responsive process. On the contrary, not much influence can be observed in the case of normal cells. The preliminary results reported here will shed new light on the future design and applications of multifunctional platforms for enhanced cancer therapy and synergetic PDT.

## Results and Discussion

### Preparation and characterization of MSN, MSN-HP, MSN-HP-DOX and MSN-HP-DOX@CeO_2_

**Figure 2:**
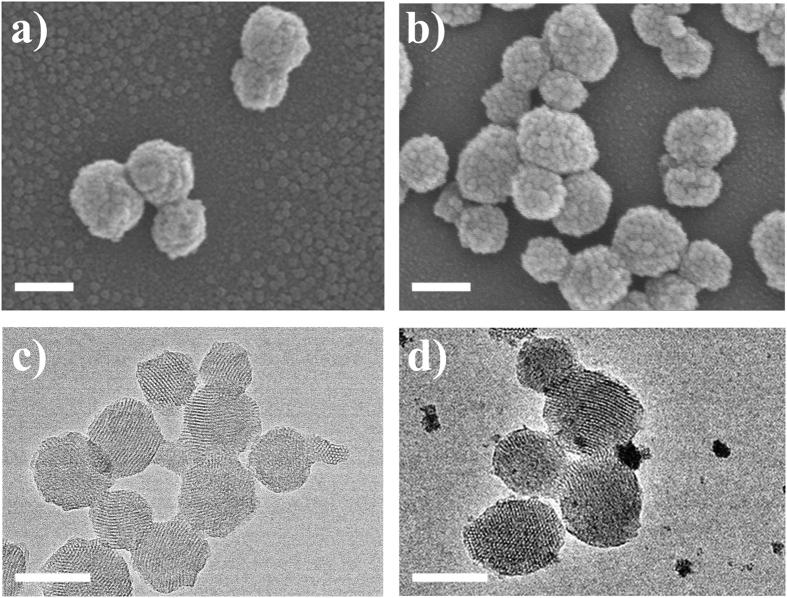
SEM micrographs of (**a**) MSN and (**b**) MSN-HP-DOX@CeO_2_. Scale bar. 100 nm; HRTEM micrographs of (**c**) MSN and (**d**) MSN-HP-DOX@CeO_2_. Scale bar: 100 nm.

MSN, MSN-HP, MSN-HP-DOX and MSN-HP-DOX@CeO_2_ were firstly prepared step by step and their physical/chemical properties were characterized in detail. Electron microscopy provides more credible information if ultrasmall nanoparticles are involved in blocking the drug loaded nanocarrier. Scanning electron microscope (SEM) images displayed highly dispersed smooth surfaced MSN, MSN-HP and MSN-HP-DOX nanospheres with a mean diameter around 100 nm (Fig. 2a and Fig. S1 in the Supporting Information). However, CeO_2_ NPs immobilization transformed the plain exterior surface of MSN into highly rough and dotted one. Large number of small and discrete dots can be observed on entire MSN surface, suggesting the complete capping of drug loaded nanochannels (Fig. 2b). In order to confirm CeO_2_ NPs anchorage, electron diffraction X-ray spectroscopy (EDX) analysis was also done which revealed the presence of elemental cerium besides silicon and oxygen signals, as can be seen in Fig. S3. In order to gain further insight, high-resolution transmission electron microscope (HRTEM) analysis was carried out, which furnished in depth information about the aggregated product. Fig. 2c and Fig. S2 illustrates that MSN, MSN-HP and MSN-HP-DOX all have a uniform and well-defined structure with vivid two-dimensional ordered channels. And the surface of these nanoparticles was smooth. However, a considerable change in MSN surface was noticed after CeO_2_ NPs immobilization. Large number of ultrasmall CeO_2_ NPs covered the outer surface (Fig. 2d).

**Figure 3:**
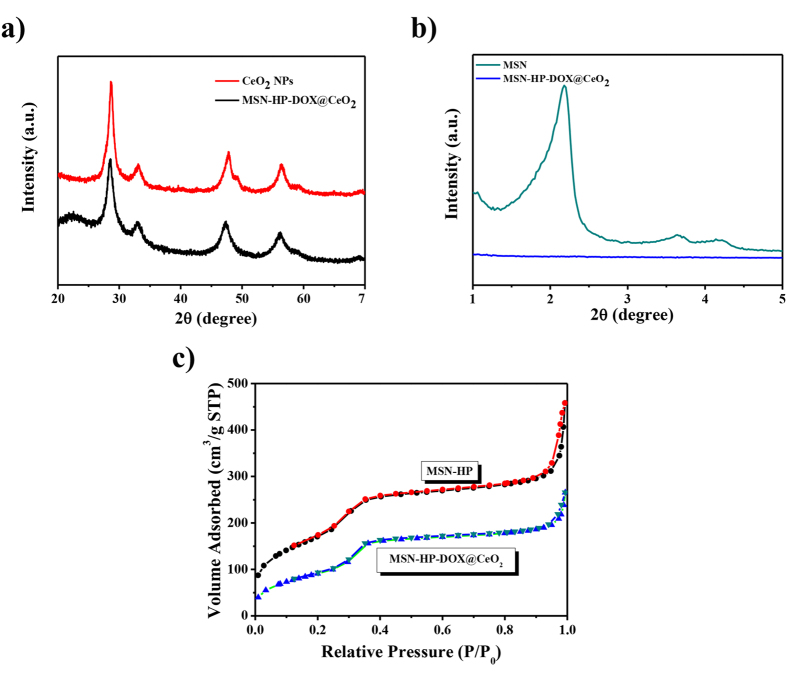
(**a**) High-angle XRD patterns of CeO_2_ NPs and MSN-HP-DOX@CeO_2_; (**b**) Low-angle XRD patterns of MSN and MSN-HP-DOX@CeO_2_; (**c**) Nitrogen adsorption−desorption isotherms for MSN-HP and MSN-HP-DOX@CeO_2_.

Furthermore, the composition and surface modifications of CeO_2_ NPs were determined through X-ray diffraction (XRD) analysis, nitrogen adsorption surface analysis and fourier transform infrared (FTIR) spectrophotometer. The purity and crystallinity of CeO_2_ NPs was identified via analyzing the XRD patterns of powdered samples. XRD patterns of MSN-HP-DOX@CeO_2_ (Fig. 3a) demonstrated a cubic phase of CeO_2_ (JCPDS card number 34–0394), while broadness of corresponding diffraction peaks suggested the formation of ultrasmall ceria nanoparticles. Low-angle XRD also provided evidence regarding the immobilization of CeO_2_ onto MSN−HP-DOX surface. Figure 3b demonstrates a reduction in the intensity of characteristic MCM-41 (100) XRD peaks after drug loading and channels capping of MSN. Nitrogen adsorption surface analysis is consistently used to characterize porous nanomaterials. Following CeO_2_ capping, BET surface area were decreased from 637.89 to 347.59 m^2^/g and pore volume were decreased from 0.665 to 0.438 cm^3^/g, respectively (Fig. 3c). FTIR spectra (Fig. S4) indicated a lack of free (i.e., non-hydrogen bonded) silanol groups in the MSN which were expected to occur at 3737 cm^−1^ 59. A broad absorption feature was observed from 3500 to 3000 cm^−1^ which was attributed to water and H-bonded silanols^59^. The narrow absorption band at 2980 cm^−1^ was due to CH stretching of ethoxy groups and/or ethanol. Absorption due to water was observed at 1630 cm^−1^ 59. Absorption at 1460 cm^−1^ was attributed to CH stretching and/or the presence of NH_4_^+^ ions^60^,^61^,^62^,^63^,^64^,^65^. Structural SiOSi absorption bands were observed at 1050 cm^−1^ 60,61,62,63,64,65. Another absorption band was present at 950 cm^−1^ due to silanols and possibly SiO- groups. After coating CeO_2_ NPs, absorption bands were decreased but still present due to the presence of CH groups (2980 cm^−1^). Meanwhile, a decrease in absorbance at 1460 cm^−1^ was indicative of loss of some NH_4_^+^ or ethoxy groups. Finally, a shift in the SiOSi absorption bands was also observed, which indicated structural change, such as further condensation. As the literature reported^60^,^61^,^62^,^63^,^64^,^65^, the removal of some NH_4_^+^ ions or ethoxy groups may be important to allow for ceria deposition on the silica surface. And specific types of silanol groups may be needed for ceria deposition on the surface.

Optical properties of MSN, MSN-HP, MSN-HP-DOX and MSN-HP-DOX@CeO_2_ were determined using UV-visible spectrometer and fluorescence spectroscopy. It can be seen in Fig. S5 that an apparent absorbance peak at about 390 nm was appeared for MSN-HP, MSN-HP-DOX which can be ascribed for HP, demonstrating the successful loading of HP; And a broad peak around 500 nm was appeared for MSN-HP-DOX which can be ascribed for DOX, demonstrating the successful loading of DOX; Moreover, a prominent absorbance peak at about 290 nm was appeared for MSN-HP-DOX@CeO_2_, which can be ascribed to the charge-transfer transitions from O 2p to Ce 4 f^60^,^61^,^62^,^63^,^64^,^65^. As shown in Fig. S6, MSN-HP exhibited the characteristic emission of HP peak around 630 nm; MSN-HP-DOX also exhibited the characteristic emission peak of DOX around 575 nm; While, not much fluorescence can be observed after CeO_2_ coating due to the fluorescence quenching ability of CeO_2_ NPs. However, upon the addition of GSH and light irradiation, the characteristic emission peak of DOX appeared again.

### GSH, pH and light irradiation triple-triggered DOX release kinetics

**Figure 4:**
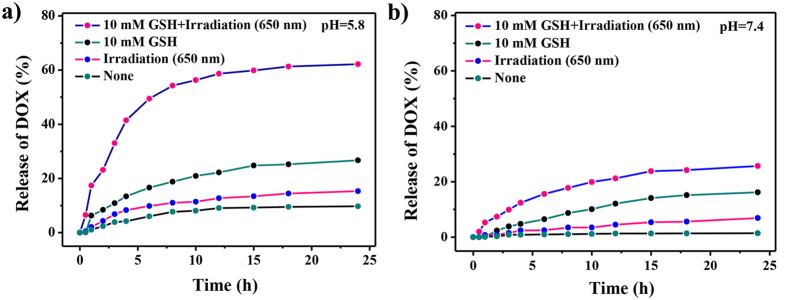
Release kinetics of DOX from MSN-HP-DOX@CeO_2_ in the presence and absence of GSH and irradiation in PBS buffer (**a**) pH = 5.8; (**b**) pH = 7.4. All data were obtained from duplicate experiments (n =3).

To investigate the feasibility of the GSH, pH and light irradiation triple-triggered release of the nanosystem, *in vitro* stimuli-responsive release of the preloaded DOX was investigated. The MSN-HP-DOX@CeO_2_ nanoparticles was added into PBS (pH 5.8 and pH 7.4) in the presence or absence of GSH (10 mM) and light irradiation, and its release efficiency was evaluated by recording absorbance at 488 nm, respectively. At pH 5.8 (Fig. 4a), which mimics the acidic environment of tumor sites, drug release rate was significantly increased and further enhancement was detected when in combination with GSH or light irradiation or GSH and light irradiation owing to the conformational change of HP destroying the combination of HP- DOX upon light irradiation and the degradation of CeO_2_ in reductive environment. While under the same conditions at pH 7.4, drug release rates were all relatively lower (Fig. 4b), suggesting that lack of anyone of reduced GSH, low pH and light irradiation, the drug release rate would be affected. Taken together, we were able to generate simple nanoparticles, MSN-HP-DOX@CeO_2_, which showed a remarkable increase in drug release in acidic environment with reduced GSH and light irradiation compared that in neutral environment owing to the benefits of triple control-release.

### Generation of singlet oxygen by the photoirradiation of MSN-HP-DOX@CeO_2_

**Figure 5:**
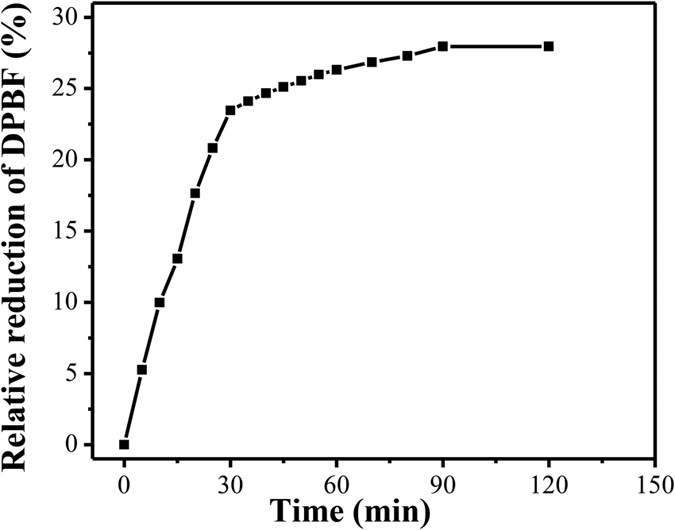
Relation between the relative loss of DPBF and light irradiation time.

In a PDT process, absorption of light by photosensitizers eventually results in generation of ^1^O_2_ and other reactive oxygen species (ROS). ^1^O_2_ is considered to be the main cytotoxic agent in PDT, and hence we have examined the efficiency of the photosensitized ^1^O_2_ generation in our nanosystem^66^,^67^,^68^. To assess the capability of ^1^O_2_ generation of MSN-HP-DOX@CeO_2_ nanoparticles, 1,3-diphenylisobenzofuran (DPBF) was employed as a probe molecule. The photo-oxidation of DPBF was monitored for 2 h under the light irradiation with a diode laser at 630 nm (Fig. S7), a typical wavelength used in PDT^68^. In the presence of MSN-HP-DOX@CeO_2_, the DPBF absorption decreased continuously over the course of light irradiation, confirming the generation of ^1^O_2_. Figure 5 shows the relationship between the relative loss of DPBF and light irradiation time in the presence of the MSN-HP-DOX@CeO_2_. These results indicate that this nanosystem can be good candidate for potential PDT.

### Intracellular imaging

**Figure 6:**
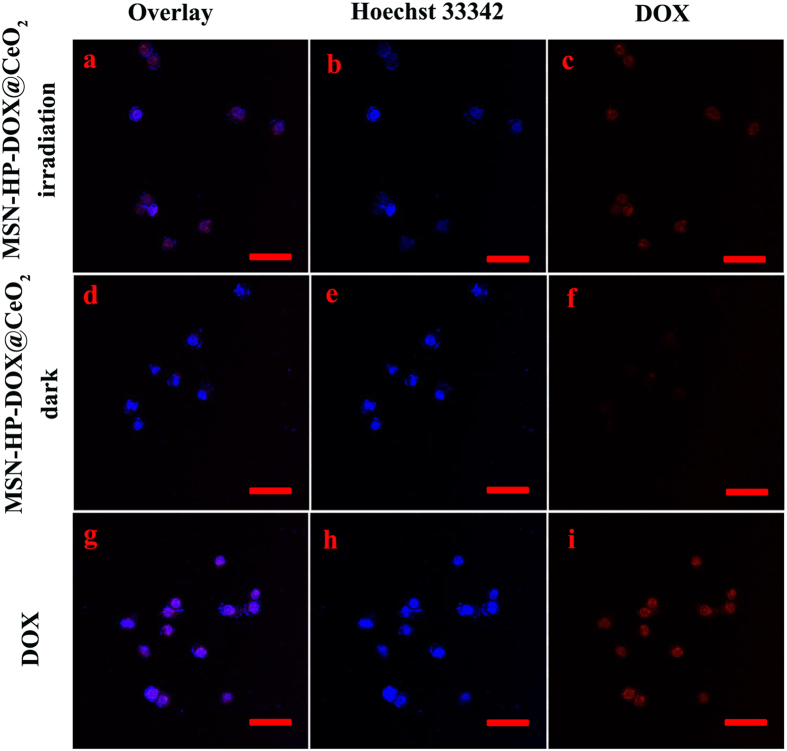
CLSM images of HeLa cells incubated with MSN-HP-DOX@CeO_2_ accompanied with light irradiation (**a–c**), MSN-HP-DOX@CeO_2_ without light irradiation (**d**–**f**) and DOX (**g–h**): panels b, e and h are blue channels with Hoechst 33342; panel c, f and i are red channels for DOX; panels a, d and g are merged images of panels b,c, e-f and h,i, respectively. Scale bar: 50 μm.

**Figure 7:**
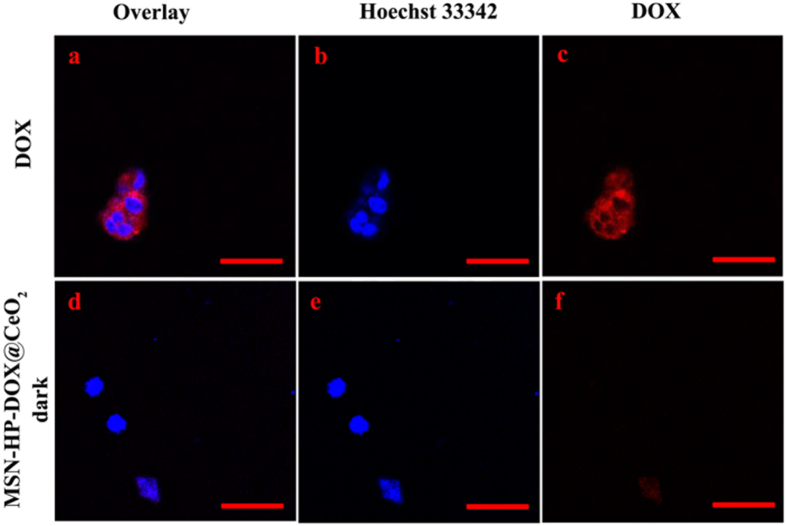
CLSM images of 293 T cells incubated with DOX (**a–c**) and MSN-HP-DOX@CeO_2_ (**d–f**): panels b and e are blue channels with Hoechst 33342; panel c and f are red channels for DOX; panels a and d are merged images of panels b,c and e,f, respectively. Scale bar: 50 μm.

To investigate intracellular applications of the triple-stimuli-responsive nanosystem, HeLa cells were separately incubated with MSN-HP-DOX@CeO_2_ with and without light irradiation and 293 T cells were incubated with MSN-HP-DOX@CeO_2_ without light irradiation. And HeLa and 293 T cells incubated with DOX alone were for comparison. MSN-HP-DOX@CeO_2_ with light irradiation was found to release DOX by the HeLa cells (Fig. 6a–c). In contrast, much less fluorescence of DOX was found to be internalized by HeLa cells incubated with MSN-HP-DOX@CeO_2_ without light irradiation (Fig. 6d–f), neither for those 293 T cells incubated with MSN-HP-DOX@CeO_2_ with and without light irradiation (Fig. 7d–f). These results strongly demonstrate that the intracellular DOX fluorescent signal was triggered by the reduction of the nanosystem’s surface CeO_2_ NPs via the combination of relative high levels GSH and acidic environment as well as the conformational change of HP triggered by light irradiation. Meanwhile, it is obvious that MSN-HP-DOX@CeO_2_ nanoparticles accompanied with light irradiation showed high selectivity for HeLa cells and the release of DOX can be monitored by “off-on” fluorescence, which is favorable for onsite detection and diagnose of cancer.

### Selective toxicity

**Figure 8:**
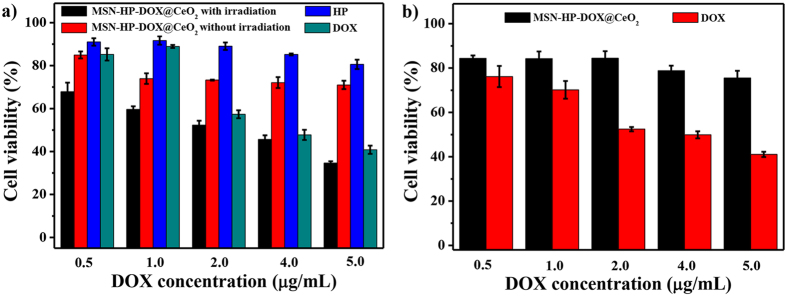
(**a**) Cytotoxicity of MSN-HP-DOX@CeO_2_ with and without radiation and HP, DOX at different concentrations on HeLa cells for 24 h. (**b**) Cytotoxicity of MSN-HP-DOX@CeO_2_ and DOX at different concentrations on 293 T cells for 24 h.

Further, the therapeutic effects of the triple stimuli-responsive nanosystem were investigated by employing MTT assay to measure the cell viability. HeLa cells were separately incubated with MSN-HP-DOX@CeO_2_, free DOX and free HP with and without light irradiation under five different concentrations for 24 h (Fig. 8a). And 293 T cells were separately incubated with MSN-HP-DOX@CeO_2_ and free DOX under five different concentrations for 24 h (Fig. 8b). As shown in Fig. 8a, for HeLa cells, the relative viabilities after the incubation with MSN-HP-DOX@CeO_2_ accompanied light irradiation were visibly lower than other three groups owing to the release of DOX under reduced GSH, pH and light irradiation stimulus and the generation of ^1^O_2_. In contrast, for 293 T cells, free DOX showed almost the same cytotoxicity with HeLa cells however MSN-HP-DOX@CeO_2_ had almost no cytotoxicity owing to the none or low release of DOX without reduced GSH, acid environment and light irradiation. These results, as expected, indicated that MSN-HP-DOX@CeO_2_ offered specific high cytotoxicity to cancer cells.

## Methods

### Reagents and apparatus

Tetraethoxysilane (TEOS), cetyltrimethylammonium bromide (CTAB), N,N-Dicyclohexylcarbodiimide (DCC), 3-aminopropyl-trimethoxysilane (APTS) 3-[4, 5-dimethylthialzol-2-yl]−2, 5-diphenyltetrazolium bromide (MTT), 1,3-diphenylisobenzofuran (DPBF) and hematoporphyrin (HP) were obtained from Sigma-Aldrich. Doxorubicin (DOX) was purchased from Aladdin (Shanghai, China). HeLa cells and 293 T cells were maintained in Dulbecco’s Modified Eagle’s Medium (DMEM, Invitrogen) with10 v/v% FBS (Fetal bovine serum), 1 v/v% penicillin/streptomycin. All other chemicals were of analytical grade and used without further purification. All aqueous solutions were prepared with ultrapure Milli-Q water (ρ > 18.0 MΩ cm^−1^).

X-ray diffraction (XRD) analysis was carried out with a D/Max2550VB+/PC X-ray diffractometer with Cu Kα (λ = 0.15406 nm), using an operation voltage and current of 40 kV and 30 mA, respectively. The absorption and emission spectra were collected using a Shimadzu 1750 UV-visible spectrometer and a RF-5301 fluorescence spectrometer (Japan), respectively. Fourier transform infrared (FTIR) spectra were obtained on a Brucher EQUINX55 FTIR spectrophotometer by a standard KBr disk method in the range 400–4000 cm^−1^. A Quanta 200 environmental scanning electron microscope (SEM) was used to observe the morphologies of the obtained materials. High-resolution transmission electron microscope (HRTEM) images and the electron diffraction X-ray spectroscopy (EDX) were recorded with a JEM-3010 transmission electron microscope operating at 200 kV. And Specimens which were prepared through dispersing the samples into alcohol via ultrasonic treatment dropped on carbon-copper grids for observation. The nitrogen adsorption and desorption isotherms were measured at liquid N_2_ temperature using a Quantachrom Autosorb-iQ, after degassing samples for 12 h at 120 °C. Surface area was calculated according to the conventional BET method and then the adsorption branches of the isotherms were used to calculate the pore parameters using the BJH method. Cell culture was carried out in an incubator with a humidified atmosphere of 5% CO_2_ at 37 °C. Cell toxicity tests were tested by microplate reader (KHB ST-360). The confocal laser microscope data were acquired using a confocal fluorescence microscope (Nikon A1R).

All of the experiments were performed in compliance with the relevant laws and institutional guidelines, and were approved by Northwest A&F University.

### Preparation of MSN and HP-conjugated MSN

Briefly, reagents of 1.0 g N-cetyltrimethyl- ammonium bromide (CTAB) and 0.28 g NaOH were dissolved into 480 mL double-distilled water and heated up to 80 °C. Then, 5 g tetraethoxysilane (TEOS) was dropwisely added for 2 h under vigorous stirring until white precipitant was formed. After centrifugation, the solid crude product was rinsed with excessive double-distilled water and methanol, respectively.

To further remove the surfactant template of CTAB, the crude product was refluxed with methanolic solution composed of 7 mL HCl (37.4%) and 120 mL methanol at 80 °C for 24 h. The resulting product was filtered, washed extensively with double-distilled water and methanol to remove the remaining solvents under high vacuum (<1000 Pa). Finally, the sample was dried in air to yield the as-synthesized MSN.

To prepare HP-conjugated MSN, DCC was used as the condensing agent to ensure that the carboxylic group of HP selectively reacts with the amino group of APTS. 100 mg of HP, 34 mg of DCC, and 1 mL of APTS were sequentially added into 3.0 mL anhydrous toluene. The mixture was stirred overnight at room temperature. The obtained HP-APTS was directly used without further treatment. Next, 1.0 g of MSNs was refluxed with 80 mL of anhydrous toluene containing 0.75 mL of HP-APTS for 20 h. The sample was donated as MSN-HP.

### Loading of DOX in MSN-HP

DOX was dissolved in ethylene glycol at 5 mM and then mixed with 5 mL MSN-HP at the concentration of 60 mg/mL. Next, the mixture was stirred for 1 h and then standing overnight. Free drug was removed by centrifugation. The drug-loading efficiency was estimated to be approximately 21.8 μg/mg by UV-*vis* spectrum. The sample was donated as MSN-HP-DOX.

### Preparation of CeO_2_ coated MSN-HP-DOX

Typically, 300 mg of dried MSN-HP-DOX were dispersed in 5 mL of ethylene glycol with ultrasonication. Cerium nitrate (1 M, 2.25 mL) was added and the mixture was stirred for 10 min. This mixture was heated to 130 °C for 15 h and then cooled to room temperature^69^. The as-prepared MSN-HP-DOX@CeO_2_ particles were isolated by centrifugation (12,000 RCF, 10 min) and washed with ethanol.

### *In Vitro* DOX release kinetics

To determine the kinetics of DOX release from MSN-HP-DOX@CeO_2_, 300 μL MSN-HP-DOX@CeO_2_ (2 mg/mL) were incubated in 2 mL of PBS buffer (pH 7.4 or pH 5.8) with and without the addition of 10 mM GSH and red light (650 nm) irradiation separately for different periods of time. Supernatant was collected by centrifugation at predetermined time points. DOX release was determined by measuring the absorption intensity at 480 nm by UV-*vis* spectrometer.

### Evaluation of ^1^O_2_

A solution of DPBF (300 μM) in DMSO (500 μL) was added into the solution of MSN-HP-DOX@CeO_2_ (0.75 mg/mL) in PBS buffer (pH 5.8, 2 mL) with 10 mM GSH. The resulting solutions containing 1.5 mg MSN-HP-DOX@CeO_2_ and 75 μM DPBF were photoirradiated with red light (650 nm) irradiation for different periods of time. And changes in the UV-*vis* spectra of DPBF were recorded.

### Confocal fluorescence microscopy

HeLa or 293 T cells were seeded in 35-mm dishes. For the free DOX group, the cells were treated with 2 μg/mL of DOX for 4 h, and then kept in the dark or white light irradiated (1.6 mW/cm^2^) for 20 min. For the MSN-HP-DOX@CeO_2_ group, MSN-HP-DOX@CeO_2_ (DOX amount was 2 μg/mL) with and without the addition of GSH (10 mM) were added to the dishes, after 4 h incubation, cells were either kept in the dark or white light irradiated (1.6 mW/cm^2^) for 20 min. The cells were then washed with PBS twice. The red fluorescence of DOX was observed after radiation by confocal scanning microscopy (Nikon A1R) with an excitation wavelength of 488 nm and emission wavelength of 560 nm.

### *In vitro* toxicity testing for MSN-HP-DOX@CeO_2_

For cell viability study, HeLa or 293 T cells were seeded in 96-well plate at a density of 5.0 × 10^3^ cells per well and cultured for 24 h. Then culture medium was replaced by 200 μL of DMEM with FBS containing various concentrations of MSN-HP-DOX@CeO_2_ or the free DOX and free HP and cells were further incubated for 4 h. Then the medium were washed with PBS and replaced with DMEM medium and plates were exposed to the white light source (1.6 mW/cm^2^) for 20 min. After light exposure, culture medium changed to DMEM medium with 10% FBS in the dark for 20 h and then evaluated with the MTT assay. 10 μL of the stock MTT solution (5 mg/mL) in PBS were added to each well and incubated for 4 h at 37 °C. Cell viability was calculated by the absorbance at 490 nm using a microplate spectrophotometer.

## Summary

In summary, we have designed and prepared a triple-triggered (GSH, pH and light irradiation) stimuli-responsive nanosystem based on CeO_2_ coated MSN. In this nanosystem, MSN was prepared as vectors for DOX and HP, and CeO_2_ NPs was adopted as gatekeeper as well as quencher for fluorescence of DOX. Benefiting from the combination of the degration of CeO_2_ NPs via reduced GSH and low pH as well as the conformal change of HP under light irradation, the more precise release of pre-loaded DOX could be realized under the so-called triple-control. The results demonstrated that this type of nanosystem afforded high specific and enhanced therapeutic efficiency for cancer cells, which could provide a general and promising platform for early therapy of cancers.

## Additional Information

**How to cite this article**: Wen, J. *et al*. Construction of A Triple-Stimuli-Responsive System Based on Cerium Oxide Coated Mesoporous Silica Nanoparticles. *Sci. Rep.* **6**, 38931; doi: 10.1038/srep38931 (2016).

**Publisher's note:** Springer Nature remains neutral with regard to jurisdictional claims in published maps and institutional affiliations.

## Supplementary Material

Supplementary Information

## Figures and Tables

**Figure 1:**
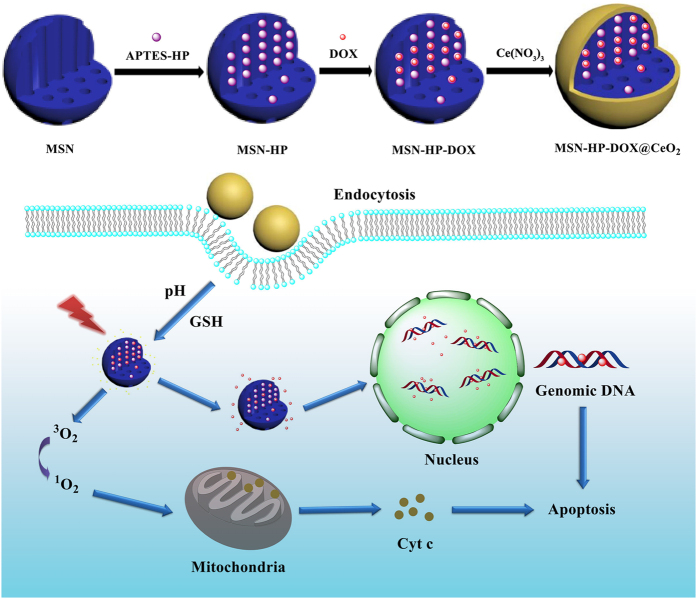
Schematic demonstration of synthetic and working protocol for triple- stimuli-responsive drug delivery system.
