# Supplementary material for: Construction of A Triple-Stimuli-Responsive System Based on Cerium Oxide Coated Mesoporous Silica Nanoparticles
Source: Sci Rep. 2016 Dec 12;6:38931. doi: 10.1038/srep38931 (PMC5150981; doi:10.1038/srep38931)
Supplement: Supplementary Information [file srep38931-s1.pdf]

Supporting Information

Construction of A Triple-Stimuli-Responsive System Based on Cerium Oxide Coated Mesoporous Silica Nanoparticles

Jia Wen [1], Kui Yang [1], Yongqian Xu [1], Hongjuan Li [1], Fengyu Liu[2,*] and Shiguo Sun[1,*]

[1]Shaanxi Key Laboratory of Natural Products & Chemical Biology, College of Science, Northwest A&F University, Yangling, Shaanxi 712100, People's Republic of China

[2]State Key Laboratory of Fine Chemicals, School of Chemistry, Dalian University of Technology, No.2 linggong Road, Ganjingzi, District, Dalian 116023, People's Republic of China

**Corresponding author:*

Fengyu Liu (liufengyu@dlut.edu.cn)

Shiguo Sun (sunsg@nwsuaf.edu.cn)

Table of contents

Fig. S1 2

Fig. S2 2

Fig. S3 3

Fig. S4 3

Fig. S5 4

Fig. S6 5

Fig. S7 5

Fig. S1

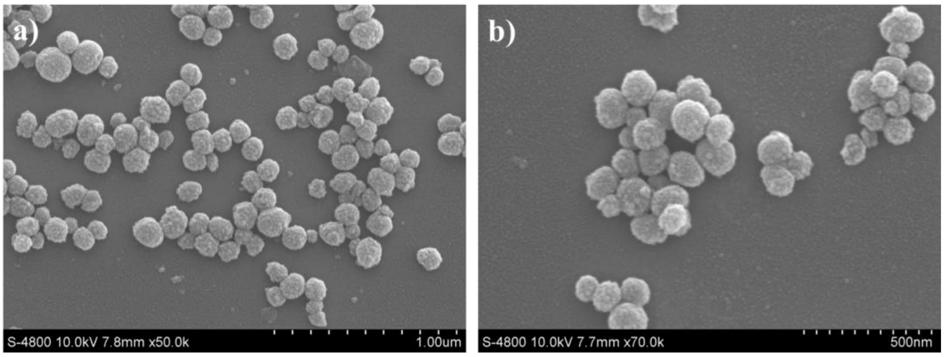

Fig. S1 SEM micrographs of (a) MSN-HP and (b) MSN-HP-DOX.

Fig. S2

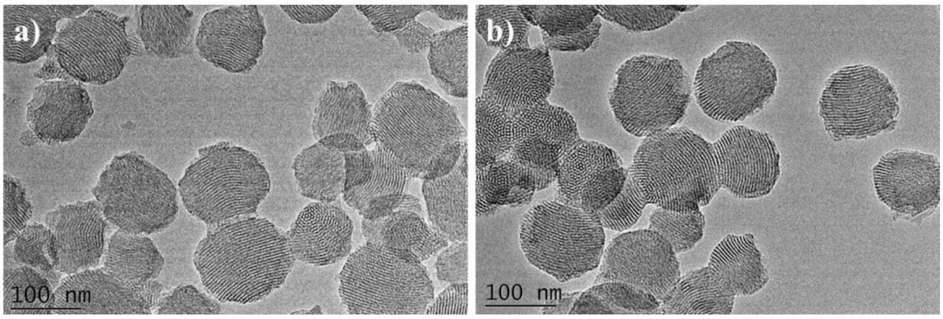

Fig. S2 HRTEM micrographs of (a) MSN-HP and (b) MSN-HP-DOX.

Fig. S3

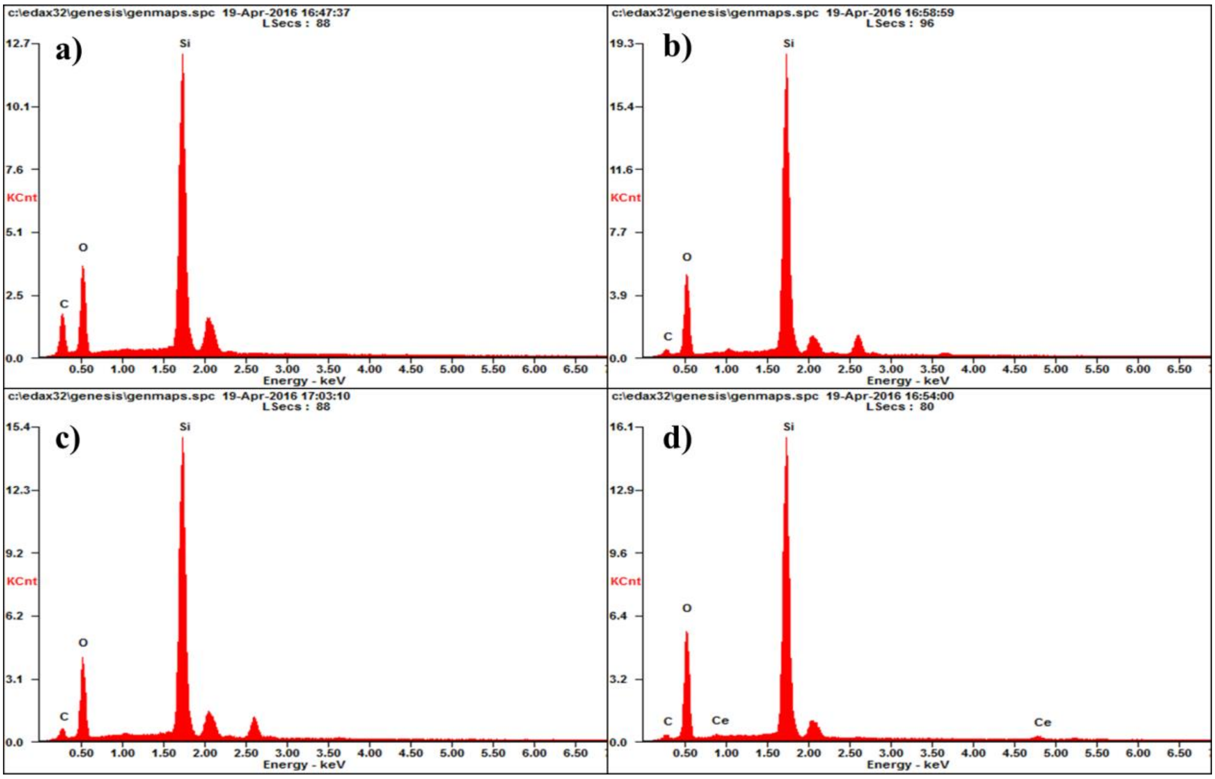

Fig. S3 EDX spectrum of (a) MSN; (b) MSN-HP ; (c) MSN-HP-DOX; (d) MSN-HP-DOX@CeO_2.

Fig. S4

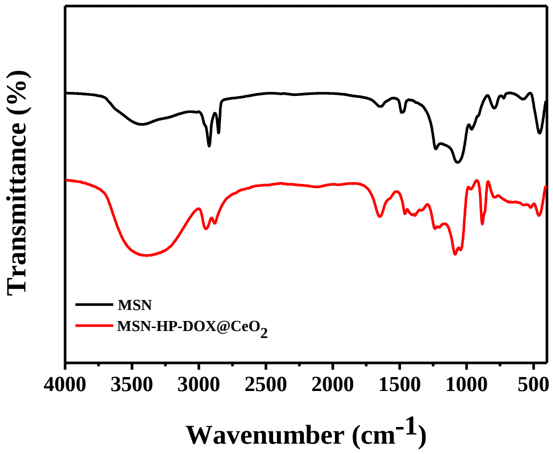

Fig. S4 FTIR spectra of MSN and MSN-HP-DOX@CeO_2.

Fig. S5

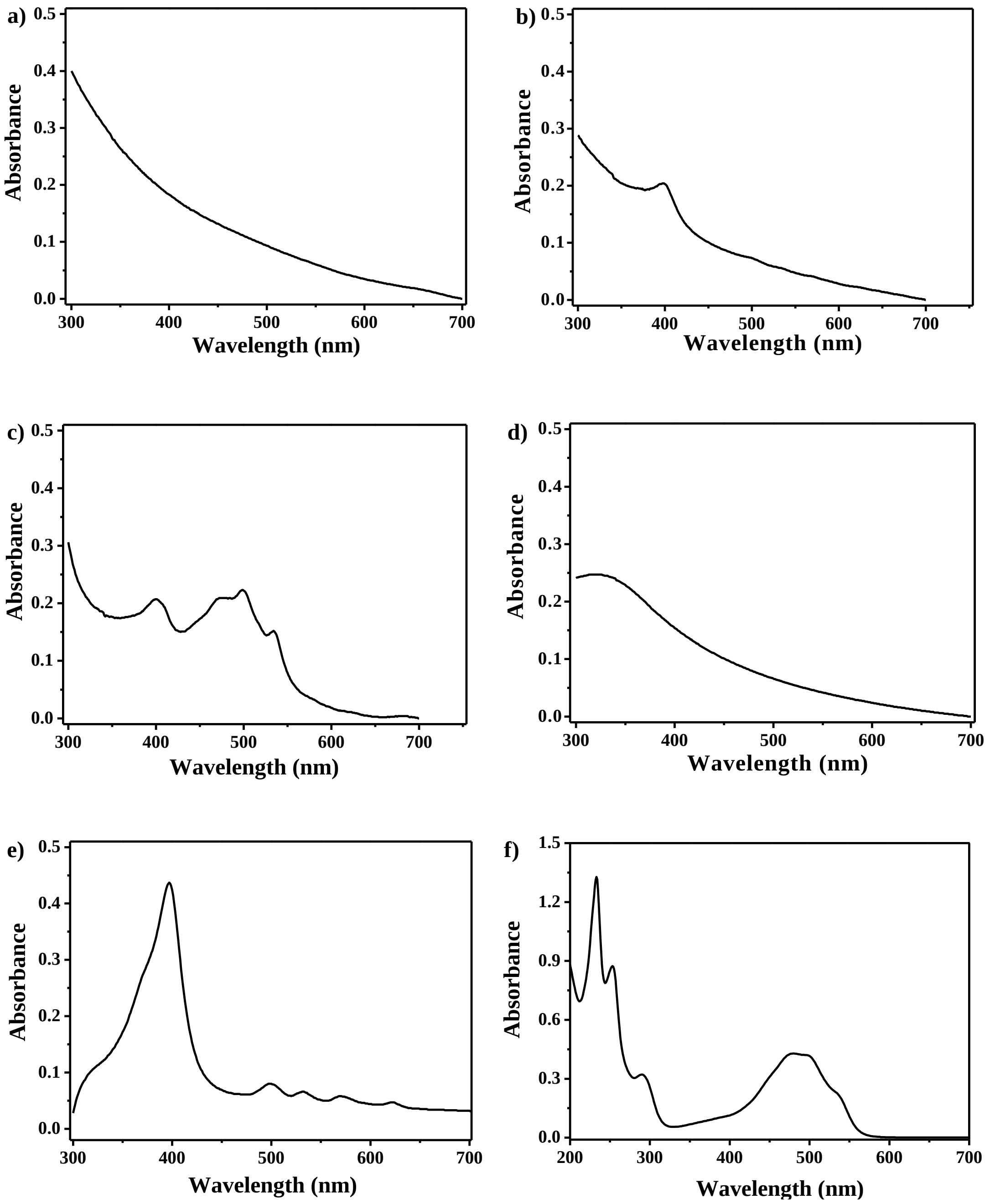

Fig. S5 Absorbance spectrum of MSN (a), MSN-HP (b), MSN-HP-DOX (c), MSN-HP-DOX@CeO_2 (d), HP (e) and DOX (f).

Fig. S6

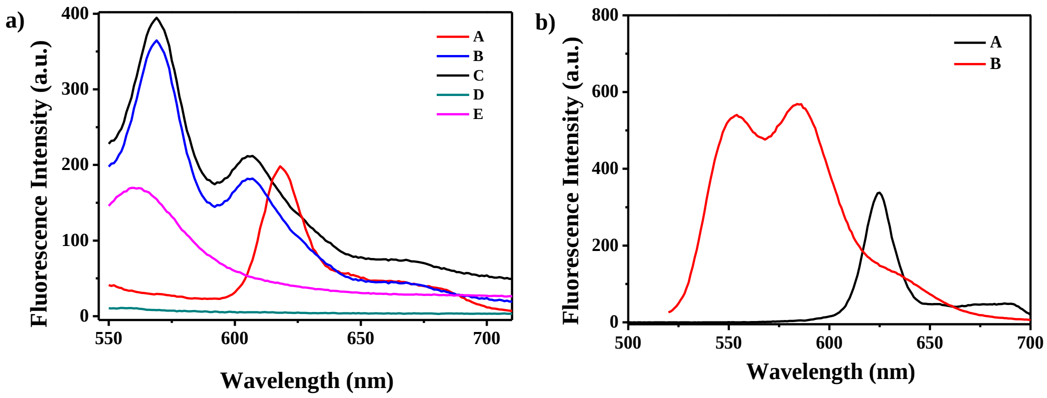

Fig. S6 (a) Emission spectra of MSN-HP (A), MSN-HP-DOX (B), MSN-HP -DOX with the addition of GSH (C), MSN-HP-DOX@CeO_2 without the addition of GSH and irradiation (D), MSN-HP-DOX@CeO_2 with the addition of GSH and irradiation (E) λ_{ex} = 480 nm. (b) Emission spectra of HP (A) and DOX (B) λ_{ex} = 480 nm.

Fig. S7

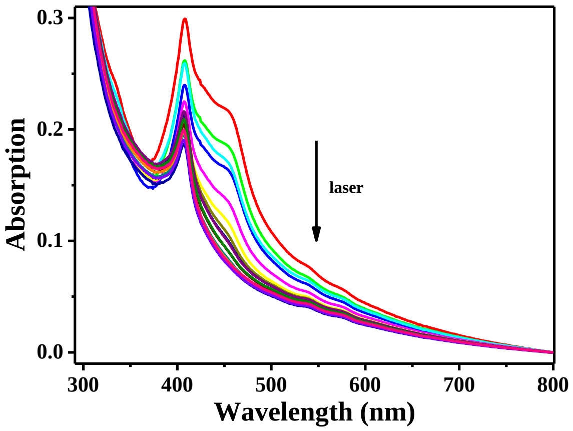

Fig. S7 Determination of the generation of singlet oxygen of MSN-HP-DOX@CeO_2 through the absorption of DPBF upon irradiation by laser at 633 nm for 0 min, 5 min, 10 min, 15 min, 20 min, 25 min, 30 min, 35 min, 40 min, 45 min, 50 min, 55 min, 60 min, 70 min, 80 min, 90 min and 120 min).
